# Rapid phase-modulated water excitation steady-state free precession for fat suppressed cine cardiovascular MR

**DOI:** 10.1186/1532-429X-10-22

**Published:** 2008-05-13

**Authors:** Hung-Yu Lin, Subha V Raman, Yiu-Cho Chung, Orlando P Simonetti

**Affiliations:** 1 Department of Biomedical Engineering, The Ohio State University, Columbus, Ohio, USA; 2 Department of Internal Medicine, Division of Cardiovascular Medicine, The Ohio State University, Columbus, Ohio, USA; 3 Dorothy M. Davis Heart & Lung Research Institute, The Ohio State University, Columbus, Ohio, USA; 4 Siemens Healthcare, Inc. Malvern, Pennsylvania, USA; 5 Department of Radiology, The Ohio State University, Columbus, Ohio, USA

## Abstract

**Background:**

The purpose of this article is to describe a steady-state free precession (SSFP) sequence for fat suppressed cine cardiovascular magnetic resonance (CMR). A rapid phase-modulated binomial water excitation (WE) pulse is utilized to minimize repetition time and acquisition time.

**Methods:**

Three different water-excitation pulses were combined with cine-SSFP for evaluation. The frequency response of each sequence was simulated and examined in phantom imaging studies. The ratio of fat to water signal amplitude was measured in phantoms to evaluate the fat suppression capabilities of each method. Six volunteers underwent CMR of the heart at 1.5T to compare retrospectively-gated cine-SSFP with and without water excitation. The ratio of fat to myocardium signal amplitude was measured for conventional cine-SSFP and phase-modulated WE-SSFP. The proposed WE-SSFP method was tested in one patient referred for CMR to characterize a cardiac mass.

**Results and discussion:**

The measured frequency response in a phantom corresponded to the numerical Bloch equation simulation demonstrating the widened stop-band around the fat resonant frequency for all water-excitation pulses tested. *In vivo *measurements demonstrated that a rapid, phase-modulated water excitation pulse significantly reduced the signal amplitude ratio of fat to myocardium from 6.92 ± 2.9 to 0.8 ± 0.13 (mean ± SD) without inducing any perceptible artifacts in SSFP cine CMR.

**Conclusion:**

Fat suppression can be achieved in SSFP cine CMR while maintaining steady-state equilibrium using rapid, phase modulated, binomial water-excitation pulses.

## Introduction

Suppression of bright fat signal is important in a variety of cardiovascular magnetic resonance (CMR) applications to characterize lesions, suppress chemical shift and motion artifacts, and distinguish fluid or tumor from adipose tissue. Numerous techniques such as chemical shift selective pre-saturation (CHESS) [1,2], short tau inversion recovery (STIR) [3,4], and the multi-point Dixon method [5] have been developed to provide suppression of signal from normal adipose tissue. These techniques all have limited success when applied to steady-state free precession (SSFP) imaging as they disturb the steady-state equilibrium and/or prolong repetition time (TR) and acquisition time. A number of recent articles describe fat suppression methods designed to maintain the magnetization steady-state in SSFP imaging [6-14]. Scheffler [7] first proposed a method of interleaving spectral fat saturation pulses within the SSFP acquisition, utilizing an α/2 flip-back pulse to store the established steady-state magnetization prior to each fat suppression pulse. While successful, this method is incompatible with cine CMR that requires continuous data acquisition without interruption. Reeder [8] proposed a water-fat separation method using an "iterative decomposition of water and fat with echo asymmetry and least squares estimation" (IDEAL) which decomposes cine-SSFP images into separate water and fat images. IDEAL requires acquisition of three complete datasets and a longer TR, nearly tripling image acquisition time and increasing sensitivity to off-resonance artifacts. Hardy [13] proposed a method of maintaining an uninterrupted, fat suppressed steady-state by cycling the SSFP RF-excitation pulse amplitude through a repeating binomial pattern. This approach utilizes the principle of binomial water-excitation [15], modulating the excitation pulse amplitudes to create a broad band of signal suppression centered on the fat frequency. However, Hardy's technique required additional TR's and a significant increase in total acquisition time. The method of alternating-TR (ATR-SSFP) proposed by Leupold [11] arrives at a similar pulse sequence design to that which we propose, but with differences in concept and in sequence design constraints that will be discussed.

A simple, practical method for spectrally and spatially selective water-excitation (WE) based on binomial pulse design [15] has been used in combination with spoiled gradient echo imaging for several years. Binomial water-excitation has been applied to abdominal and orthopedic MRI [16-18], and more recently to CMR [19] providing advantages of no disruption of the steady-state and uniform fat suppression. More recently, binomial water-excitation has been combined with 3D SSFP for orthopedic imaging [20]. In this work, we combine a rapid phase-modulated binomial water-excitation pulse with SSFP for fat suppressed cardiac cine imaging. Our hypothesis is that sufficient fat signal suppression can be achieved with minimal impact on TR, sensitivity to flow artifact, total scan time, and cine-SSFP image quality using rapid binomial water-excitation RF pulses. While the combination of binomial water-excitation with SSFP has similarities with the methods proposed by both Hardy [13] and Leupold [11], our design strategy removes the necessity for any additional data acquisition or constraints on the relationship between the TR and the water-excitation pulse timing. Numerical simulation, phantom and healthy volunteer imaging trials were performed to provide experimental validation of the fundamental concepts and performance of WE-SSFP, and images in one patient are shown to demonstrate a potential clinical application.

## Methods

### Phase-modulated water excitation

**Figure 1:**
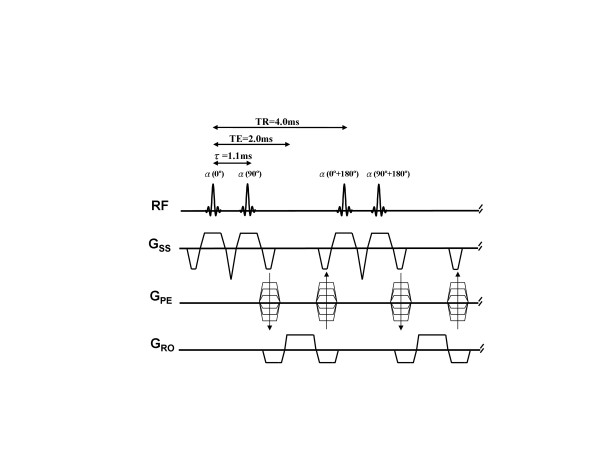
**Pulse sequence diagram for phase-modulated, binomial 1-(90°)-1 water excitation cine-SSFP.** The two consecutive α° flip angle, selective RF pulses with 90° phase increment results in an inter-pulse delay of τ = 1.1 ms for water-only excitation. Note that all gradients are fully balanced on all axes to maintain the coherent steady-state.

Spectral-spatial water excitation can be achieved using a spatially-selective RF pulse train with flip angles following a binomial series (1-1, 1-2-1, 1-3-3-1, etc.) [15]. Increasing the number of component pulses and therefore the order of the binomial pulse improves spectral selection, but at the expense of total RF pulse duration. The simplest binomial pulse (1-1) consists of two α° pulses with inter-pulse delay (τ) chosen to allow 180° of phase evolution between water and fat spins (τ = 2.2 ms at 1.5 Tesla). The first pulse rotates both fat and water magnetization toward the transverse plane. After time τ, fat and water spins are 180° out of phase and the second pulse, identical to the first in both amplitude and phase, tips water protons further down towards the transverse plane while tipping fat protons back up to the longitudinal axis. This pulse combination effectively reverses the initial excitation of fat, and the resultant tip angle for water is the sum of the individual component pulse angles. In SSFP applications, it is critical to keep the total RF pulse duration as short as possible to avoid lengthening the repetition time. Rather than waiting for 180° of phase evolution between component pulses, phase-modulated water excitation employs a partial (< 180°) off-resonance phase evolution to shorten the combined binomial pulse duration [21]. The phase of the second RF pulse is set to tip the fat magnetization back up to the longitudinal axis, and also provides some additional tip down of water. This strategy of "phase-modulated water excitation" was used to design a minimum time spatial-spectral selective binomial pulse for combination with cine-SSFP. Figure 1 shows a SSFP sequence utilizing a simple 1-1 binomial slice-selective RF pulse with 1.1 ms inter-pulse spacing to allow 90° of fat-water phase evolution (1-(90°)-1). This was found to be the minimum inter-pulse spacing necessary to accommodate the standard apodized-sinc RF pulses (600 μsec duration) used for cine-SSFP on our 1.5T MRI system (MAGNETOM Avanto, Siemens Medical Solutions, Inc. Malvern, PA).

The performance of three different binomial water excitation pulses were investigated by numerical simulation, imaging studies of water and fat phantoms, and normal volunteer imaging. Four pulses were compared: (a) conventional slice-selective apodized-sinc RF pulse, (b) spectral-spatial binomial 1-(180°)-2-(180°)-1 WE pulse with 180° phase evolution (inter-pulse delay = 2.2 ms), (c) spectral-spatial binomial 1-(180°)-1 WE pulse with 180° phase evolution (inter-pulse delay = 2.2 ms), and (d) spectral-spatial binomial 1-(90°)-1 phase-modulated WE pulse with 90° fat-water phase evolution (inter-pulse delay = 1.1 ms), and 90° phase offset between the two pulses in the 1-1 pair. The same RF pulse envelope and duration (600 μsec) were used for all individual component excitation pulses. The effective flip angle is defined as the total flip angle for on-resonant water spins. All RF pulse design and acquisition parameters are provided in Table 1.

**Table 1:** Summary of imaging parameters for phantom and *in vivo *studies*

**Sequences**	**Interpulse Phase Evolution (°)**	**Interpulse Delay (ms)**	**Total Pulse Duration (ms)**	**TR for Phantom Studies (ms)**	**TR for *in vivo *Studies (ms)**	**Component Pulse Flip Angles (°)**	**Resultant Flip Angle (°)**
**Standard SSFP**	N/A	N/A	0.6	9.68	3.1	70	70
**1-2-1 WE-SSFP**	180	2.2	5.0	9.68	8.9	17.7 – 35.4 – 17.7	70
**1-1 WE-SSFP**	180	2.2	2.8	9.68	6.5	35.4 – 35.4	70
**1-1 WE-SSFP**	90	1.1	1.7	9.68	4.0	56.4 – 56.4	70

* NA: not applicable

### Numerical simulations

Simulations were run to predict the variation of steady-state transverse magnetization with chemical shift for the SSFP sequence in combination with the four different excitation pulses. All simulations were performed with the following simulation parameters: TR = 9.68 ms, TE = 4.8 ms, Flip angle = 70° for the conventional SSFP and all WE-SSFP sequences; relaxation time constants of simulated water-based tissue (T_1 _= 578 ms, T_2 _= 263 ms) and fat (T_1 _= 252 ms, T_2 _= 81 ms) were chosen to match the phantom compartments. The TR was chosen to match that used in the phantom study of pulse sequence frequency response. The frequency response of the 1-(90°)-1 pulse was also simulated at shorter TR's (8.9 ms, 5.9 ms, and 4.45 ms) to investigate any impact of TR on the fat suppression frequency band. Analytic expressions for the resulting rotation matrices and magnetization distributions were generated using *Mathematica *(Wolfram Research, Inc., Champaign, IL.).

### Pulse sequence implementation

WE-SSFP cine sequences using each of the four pulse designs were implemented on a 32-channel, 1.5 Tesla MR system (MAGNETOM Avanto, Siemens Medical Solutions, Erlangen, Germany) with 45 mT/m gradient amplitude and 200 mT/m/ms maximum slew rate. Phantom and human imaging studies were performed using twelve array coil elements.

Table 1 shows the CMR imaging parameters used for phantom and human volunteer studies. A 2D SSFP cine with retrospective ECG-gating was used with an effective 70° total flip angle, 5-mm section thickness, a 256 × 192 acquisition matrix, and 350 × 262 mm FOV, one signal average, and parallel acquisition acceleration rate of 2 using "Generalized Autocalibrating Partially Parallel Acquisitions" (GRAPPA). These imaging parameters were held constant throughout all phantom and human imaging experiments. In phantom studies designed to demonstrate the frequency response, the TR was set long enough (9.68 ms) to allow for the longest (1-2-1) RF pulse and keep the spacing of band artifacts the same among the four sequences. In fat/water phantom and human imaging experiments, the TR was set to the minimum permitted by each sequence in order to illustrate the benefits of minimizing the RF pulse duration. The shortest water excitation pulse, 1-(90°)-1, was also tested at longer TR values in phantoms and *in vivo* to demonstrate the independence of fat suppression to choice of TR, and the loss of image quality and increased flow sensitivity as a result of longer TR.

### Phantom imaging studies

The first phantom study was performed on a uniform spherical water phantom doped with 1.25 g NiSO_4 _+ 6 H_2_O and 5 g NaCl per 1000 g water. This phantom was imaged with an applied constant gradient offset of 0.0723 mT/m in the x-direction (left-right) to demonstrate the effect of each of the four excitation pulses on the frequency response of the cine-SSFP sequence. Images were acquired using all four pulse designs and signal profiles were measured in the direction of the applied field inhomogeneity to illustrate the frequency response and compare to the simulation results. TR was kept constant at 9.68 ms across the four sequences to maintain spacing of banding artifacts for comparative purposes.

The second phantom experiment was performed using water and mineral oil phantoms (T_1_/T_2 _of water = 578/263 ms and T_1_/T_2 _of oil = 252/81 ms) to measure the ratio of fat to water signal amplitude for each pulse and compare to that expected based on simulation results. The regions of interest (ROI) measured in the phantom images were the maximum size permissible within the boundaries of the object. The SSFP sequence was tested using the shortest TR allowed by each excitation pulse scheme. Additionally, the shortest phase modulated 1-(90°)-1 pulse was tested at longer TR's (5.0 ms and 5.6 ms) to demonstrate the independence of fat-suppression from the choice of TR.

### Human subject imaging studies

Conventional cine-SSFP and three different WE-SSFP sequences were evaluated in six healthy volunteers (1 women; aged 46 years, and 5 men; aged 22-57 years, with a mean age of 43.25 ± 13.72) and with no history of common cardiovascular disease. Vertical and horizontal long-axis views were acquired in each subject using each of the four sequences. The phase modulated 1-1 WE-SSFP sequence was also tested in one 42 year-old male patient referred for CMR to characterize a cardiac mass seen on echocardiography. All images were acquired using electrocardiographic (ECG) signal gating and breath-holding. No patient-specific or volume-localized shimming was performed. The default shim values based on field homogeneity in a uniform spherical phantom were used for all *in vivo *studies. All subjects gave written informed consent to participate in this Institutional Review Board-approved protocol.

One individual (HYL) measured the signal amplitude in the myocardium and fat in all cine series acquired in normal subjects. Measurements were made in a single, end-diastolic frame from each of the cine series acquired in the two different views using each of the four sequences. Circular ROI's were placed within the left ventricular myocardium and surrounding fat to measure average signal amplitudes (SA). For consistency, similar anatomical regions were selected in all images. The signal amplitude ratio between fat and myocardium was calculated to evaluate the effect of fat-suppression.

## Results

### Numerical simulations and phantom imaging studies

**Figure 2:**
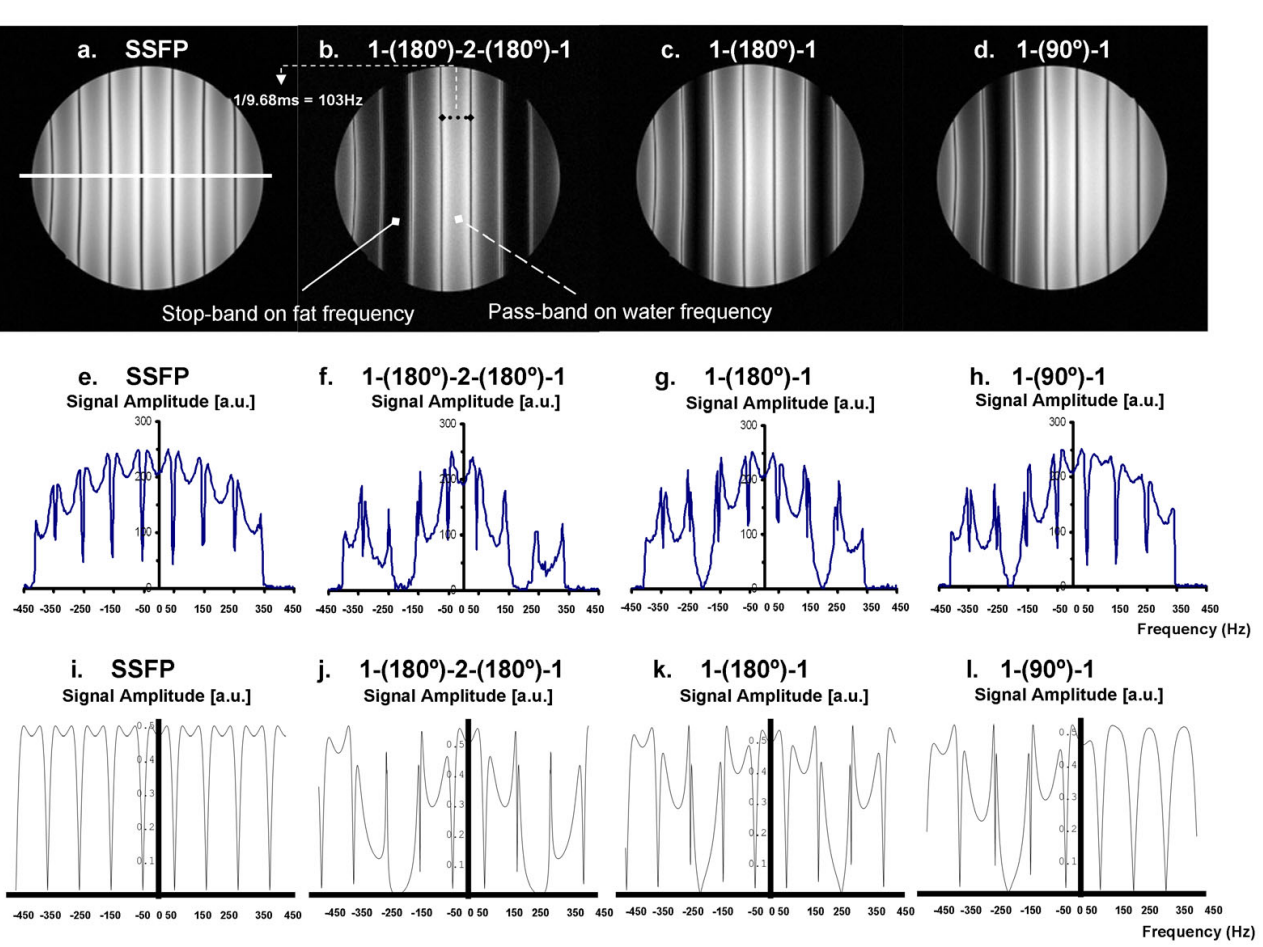
**A comparison of measured and simulated frequency response patterns for SSFP and WE-SSFP.** Top two rows demonstrate measured frequency response functions in a uniform water phantom for **(a) **conventional slice-selective RF pulse, **(b) **1-(180°)-2-(180°)-1, **(c) **1-(180°)-1, and **(d) **1-(90°)-1. All four sequences were run with TR = 9.68 ms and constant gradient offset of 0.0723 mT/m left-to-right to illustrate the signal over a range of offset frequencies. Middle row **(e-h) **shows the signal profile across the phantom for each of the corresponding images. The white line across **(a) **indicates the location of the signal profile measurement for each image. Bottom row **(i – l) **shows simulated frequency response functions for the same four sequences used to generate the phantom images **(a-d) **and signal profiles **(e-h)**. Reasonable agreement is observed between phantom measurements and simulation results.

**Figure 3:**
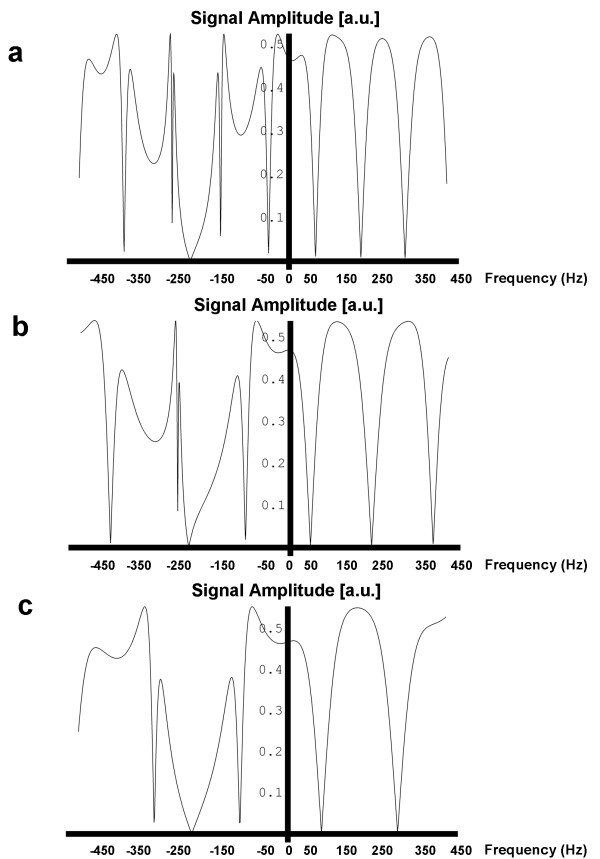
**A comparison of simulated frequency response patterns for 1-(90°)-1 WE-SSFP with **(a) **TR = 8.9 ms **(b) **TR = 5.9 ms and **(c) **TR = 4.45 ms conditions.** The fat frequency falls within the stopband in each case, indicating that fat suppression is independent of sequence TR.

**Figure 4:**
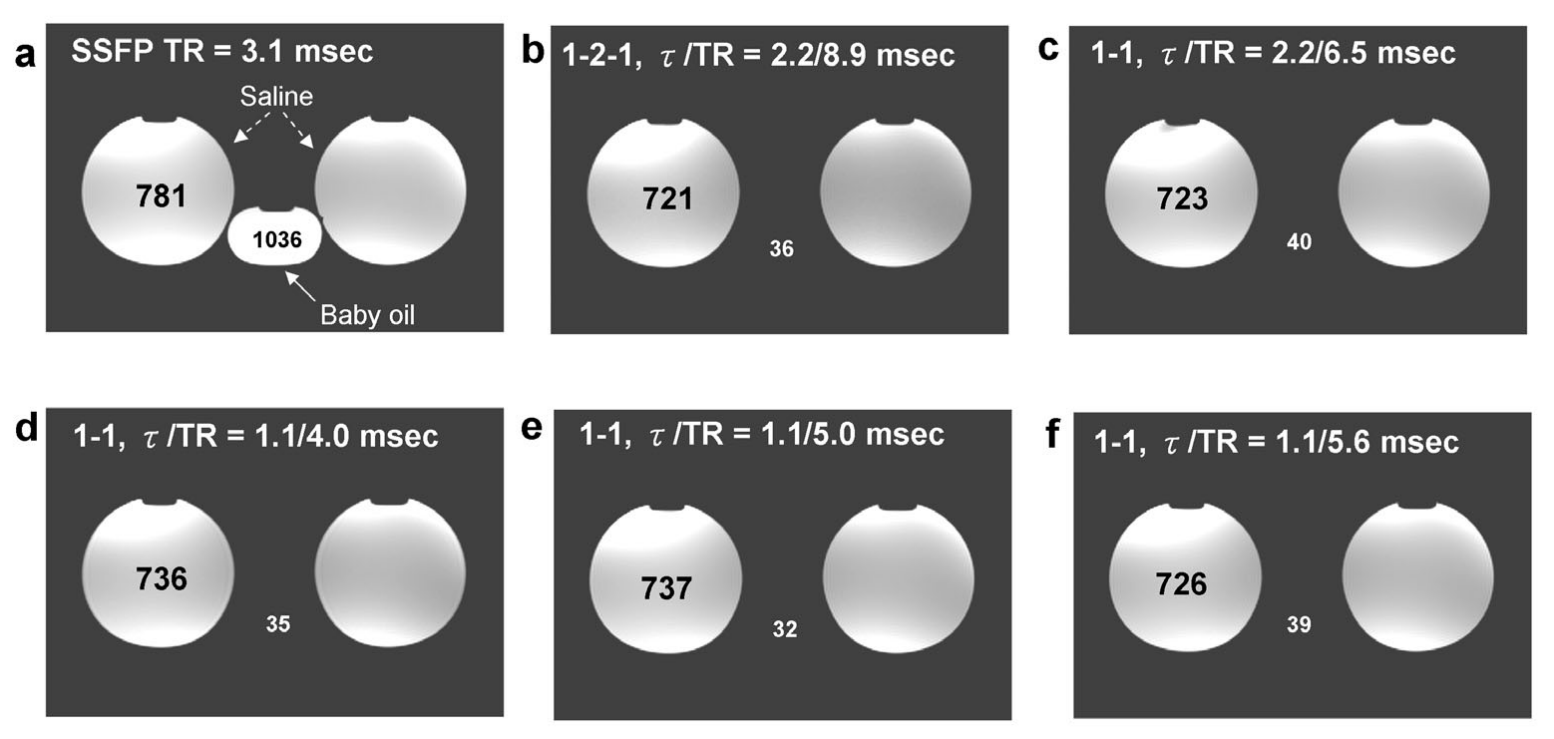
**Fat/water phantom images acquired with **(a) **conventional slice-selective RF pulse with TR = 8.9 ms, **(b) **1-(180°)-2-(180°)-1 with TR = 8.9 ms, **(c) **1-(180°)-1 with TR = 6.5 ms, and **(d) **1-(90°)-1 with TR = 4.0 ms, **(e) **1-(90°)-1 with TR = 5.0 ms, **(f) **1-(90°)-1 with TR = 5.6 ms.** WE-SSFP cine sequences show successful suppression of the fat (mineral oil) signal with maintained steady-state water signal for all binomial WE pulses over a range of TR's.

**Figure 5:**
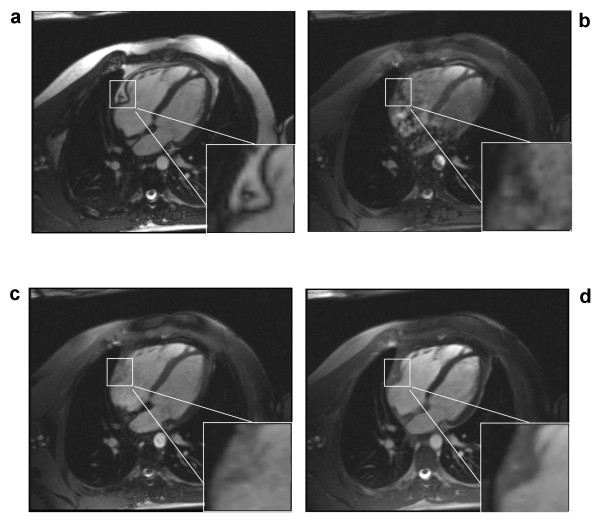
**Cardiac images acquired in a normal human subject in four-chamber view using **(a) **conventional slice-selective RF pulse with TR = 3.1 ms, **(b) **1-(180°)-2-(180°)-1 with TR = 8.9 ms, **(c) **1-(180°)-1 with TR = 6.5 ms, and **(d) **1-(90°)-1 WE-SSFP sequences with TR = 4.0 ms.** Flip Angle/Slice Thickness/Matrix = 70°/5 mm/256 × 192 for all images. A magnified region is shown in the lower right corner of each image to illustrate the signal in epicardial fat surrounding the right coronary artery in the atrioventricular groove. Significant fat signal attenuation is demonstrated by all binomial WE pulses, and no perceptible artifacts are observed in the 1-(90°)-1 WE-SSFP images. All figures are displayed with the same window and level settings.

**Figure 6:**
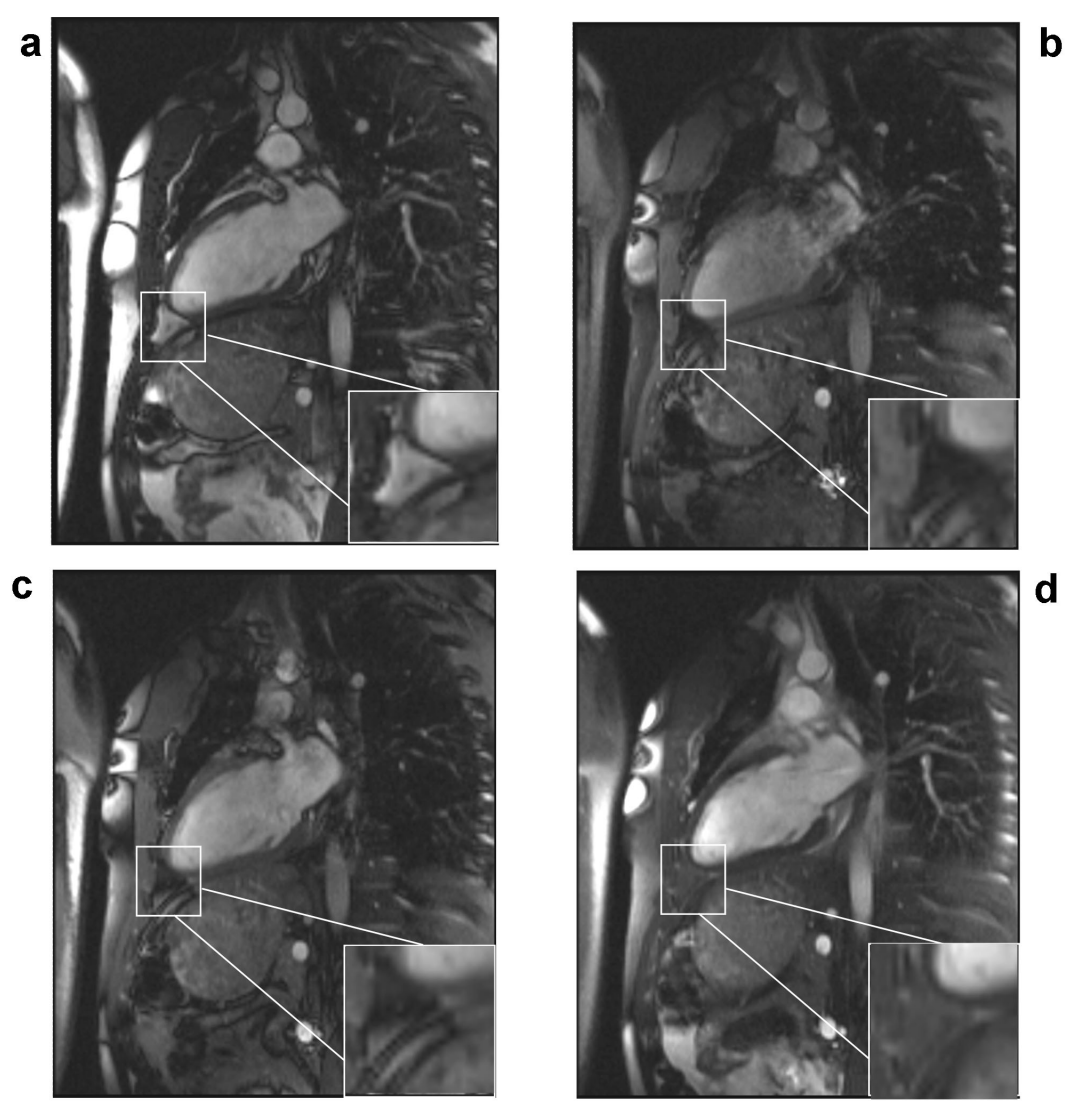
**Cardiac images acquired in a normal human subject in vertical long-axis view using **(a) **conventional slice-selective RF pulse with TR = 3.1 ms, **(b) **1-(180°)-2-(180°)-1 with TR = 8.9 ms, **(c) **1-(180°)-1 with TR = 6.5 ms, and **(d) **1-(90°)-1 WE-SSFP sequences with TR = 4.0 ms.** A magnified region is shown in the lower right corner of each image to illustrate the signal in epicardial fat surrounding the apex of the left ventricle. The phase-modulated 1-(90°)-1 WE-SSFP sequence decreases the fat to myocardium signal ratio and provides a valuable method of differentiating fluid from adipose tissue. All figures are displayed with the same window and level settings.

**Figure 7:**
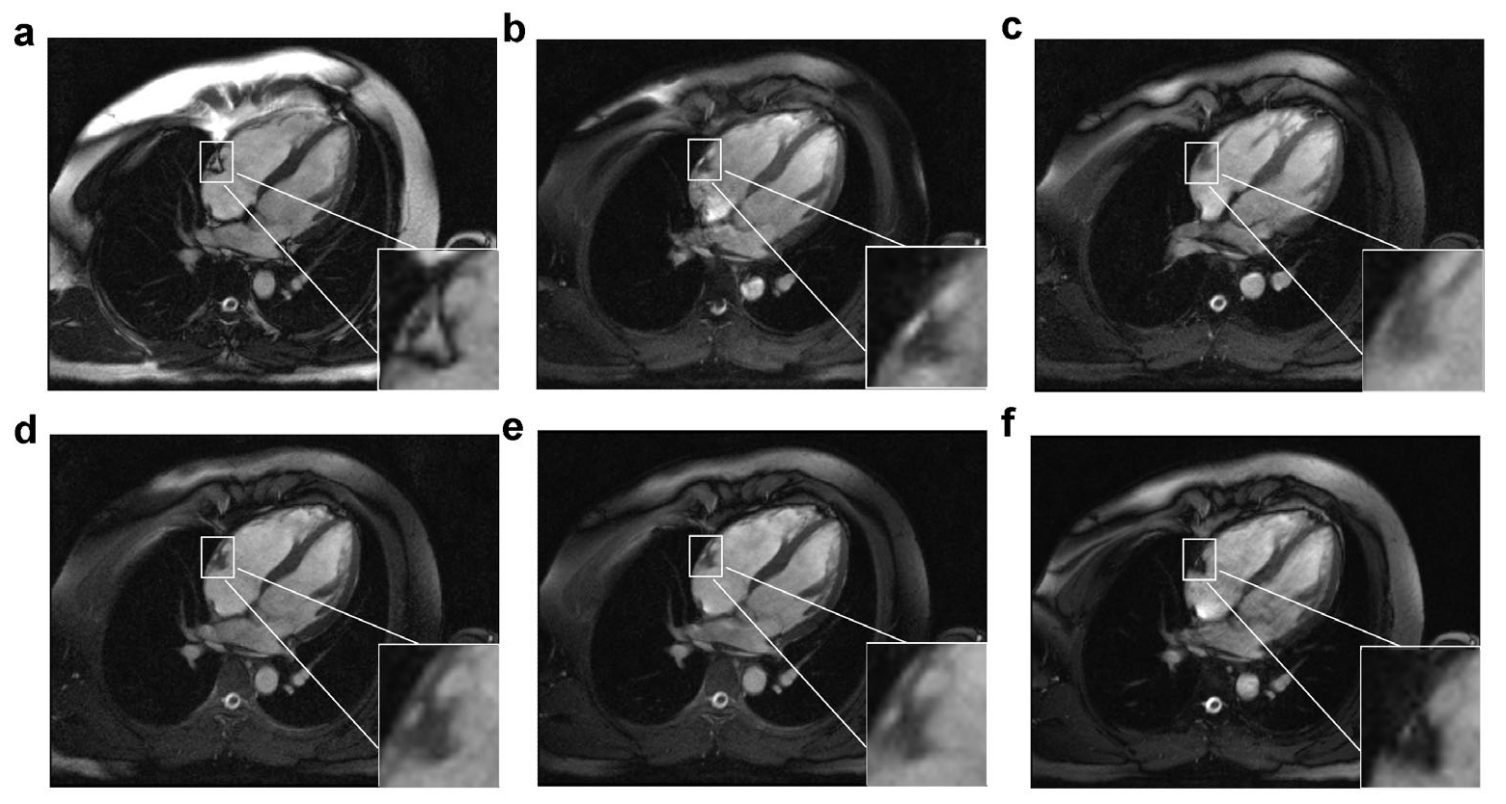
**Cardiac images acquired in a normal human subject in horizonal long-axis view using **(a) **conventional slice-selective RF pulse with TR = 3.1 ms, **(b) **1-(180°)-2-(180°)-1 with TR = 8.9 ms, **(c) **1-(180°)-1 with TR = 6.5 ms, **(d) **1-(90°)-1 and TR = 4.0 ms. **(e) **1-(90°)-1 and TR = 5.0 ms.** (f) 1-(90°)-1 and TR = 5.6 ms. These pulses and TR values correspond to those demonstrated in the phantom images shown in Figure 4. A magnified region is shown in the lower right corner of each image to illustrate the signal in epicardial fat surrounding the apex of the left ventricle. Fat suppression characteristics are seen to be independent of sequence TR. All figures are displayed with the same window and level settings.

**Figure 8:**
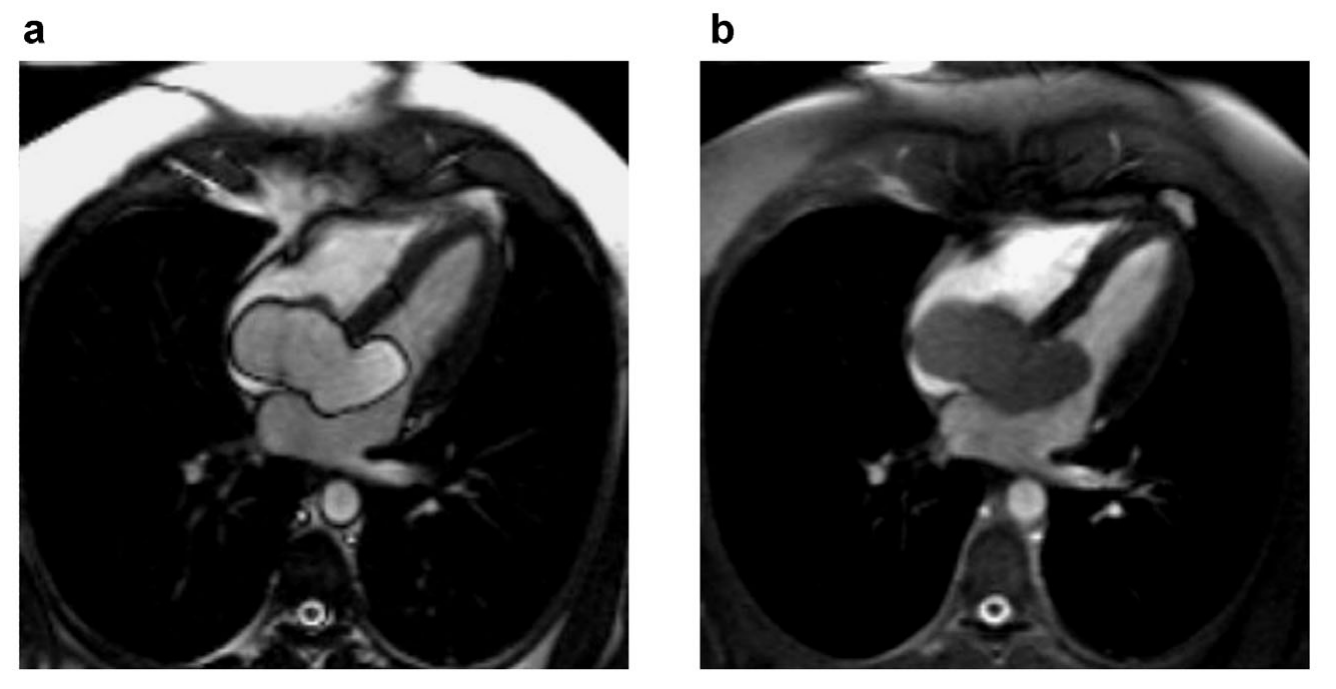
**Single end-systolic frame from cine-SSFP series acquired without **(a) **and with **(b) **phase-modulated binomial water excitation in a patient with large intracardiac lipoma.** Note the significant suppression of signal in the mass in the WE-SSFP image (b), clearly indicating this as adipose tissue. Both images are displayed with the same window and level settings.

**Figure 9:**
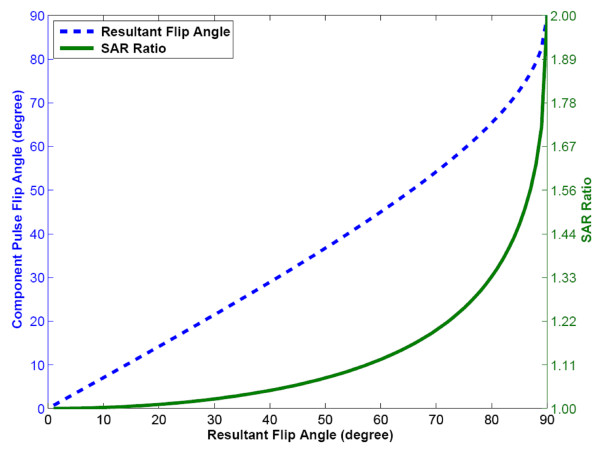
**The on-resonance (water) flip angle that results from a given component pulse flip angle is shown for the 1-(90°)-1 WE pulse.** (b) The SAR ratio of 1-(90°)-1 WE pulse compared to a conventional RF excitation of the same resultant on-resonance flip angle.

Figure 2 shows the measured frequency response profiles for SSFP with each of the four different excitation pulses (Figures 2a–d). Signal profiles measured along the direction of intentional linear field inhomogeneity are shown (Figures 2e–h) along with the results of computational Bloch equation simulations (Figures 2i–l) for comparison. For the conventional SSFP sequence (Fig. 2a, e and 2i), if TR is set exactly to 2.2 ms + n*4.4 ms (i.e., 2.2 ms, 6.6 ms, 11.0 ms, etc.), a null will be centered over the fat resonance while leaving a broad plateau over the water peak. However, this null is too narrow to suppress fat reliably. The 1-(180°)-2-(180°)-1 (Figures 2b, f, j), 1-(180°)-1 (Figures 2c, g, k) and 1-(90°)-1 (Figures 2d, h, l) binomial pulses all broaden the fat resonance stop-band and maintain the on-resonance pass-band. The measured frequency responses shown in Figures 2e–h correspond with the numerical Bloch Equation simulation results (Figures 2i–l) demonstrating the widened stop-band centered on the fat resonance. The higher signal seen in the center of the phantom is commonly observed and is due to uneven distribution of RF energy. The simulated frequency response profiles in Figure 3 demonstrate that the stopband frequency of the 1-(90°)-1 binomal pulse is centered on the fat frequency independent on the choice of TR. Phantom fat/water images presented in Figure 4 show that all tested binomial WE pulse combinations suppress the fat signal and maintain the signal amplitude of water. Phantom images obtained from the binomial 1-(180°)-2-(180°)-1 (Figure 4b), 1-(180°)-1 (Figure 4c), and phase-modulated 1-(90°)-1 (Figure 4d–f) WE-SSFP cine sequences all show successful suppression of the fat (baby oil) signal. The resulting phantom image signal measurements listed in Table 2 demonstrate that the phase-modulated 1-(90°)-1 WE pulse significantly decreased the fat to water signal ratio over a range of TR's, in agreement with the simulation results shown in Figure 3.

**Table 2:** Signal amplitude ratio between fat and water in phantom studies

**Sequences for Fat/water Studies**	**Interpulse Phase Evolution (°)**	**Interpulse Delay (ms)**	**TR (ms)**	**Water Signal**	**Fat Signal**	SA Ratio**
**Standard SSFP**	NA	NA	8.9	781 ± 27.1	1036 ± 32.6	1.327
**1-2-1 WE-SSFP**	180	2.2	8.9	721 ± 24.3	36 ± 10.8	0.050
**1-1 WE-SSFP**	180	2.2	6.5	723 ± 25.7	40 ± 11.2	0.055
**1-1 WE-SSFP**	90	1.1	4.0	736 ± 25.2	35 ± 9.8	0.048
**1-1 WE-SSFP**	90	1.1	5.0	737 ± 21.3	32 ± 8.7	0.043
**1-1 WE-SSFP**	90	1.1	5.6	726 ± 20.8	39 ± 10.4	0.054

* NA: not applicable.

** SA ratio: signal amplitude between fat and water: SA_Fat_/SA_Water_

### Human subject imaging studies

A conventional cine-SSFP image is shown in Figure 5a along with results from the 1-(180°)-2-(180°)-1 (Figure 5b), 1-(180°)-1 (Figure 5c) and the phase-modulated 1-(90°)-1 pulse (Figure 5d). These images were acquired at the minimum TR permitted by each of the pulses. All binomial WE pulses show marked fat signal reduction compared to conventional cine-SSFP. The uniformity of fat suppression was best using the 1-(180°)-2-(180°)-1 pulse (Figure 5b), as expected since it has the broadest stopband as shown in the simulation and phantom results. However, severe field inhomogeneity artifacts and flow artifacts appear most likely because this lengthy excitation pulse requires an impractically long TR (8.9 ms). Artifacts are reduced in images acquired using the shorter TR possible with the 1-1 pulses with full (Figure 5c) or partial (Figure 5d) phase evolution. The phase-modulated 1-(90°)-1 pulse demonstrates an appreciable degree of fat suppression with only a 29% increase in TR (3.1 ms vs. 4.0 ms) without any noticeable artifacts due to flow or field inhomogeneity. Magnifications of the atrioventricular groove shown in the lower right corner of each image in Figure 5 demonstrate the successful suppression of epicardial fat by the phase-modulated 1-(90°)-1 excitation pulse. However, fat is not as uniformly suppressed throughout the field-of-view as with the 1-(180°)-2-(180°)-1 pulse (Figure 5b), probably due to the narrower stop-band demonstrated in Figure 2. Figure 6 shows the lipid signal is well attenuated in all WE methods and artifact free images are generated by the phase-modulated 1-(90°)-1 cine-SSFP sequence in a vertical long-axis view of the heart of a second normal subject. Signal measurements in *in vivo *studies demonstrated that phase-modulated 1-(90°)-1 WE-SSFP significantly reduced the fat-myocardium signal amplitude ratio from 6.92 ± 2.9 to 0.8 ± 0.13 with minimal increase in TR and without inducing any perceptible artifacts. In Figure 7, conventional cine-SSFP, 1-2-1, 1-1 and phase modulated 1-1 with a variety of TR's from 4.0 to 5.6 ms are displayed in a vertical long-axis view in a normal human subject. The consistency of fat signal attenuation demonstrates that fat suppression with phase-modulated 1-(90°)-1 water excitation is independent of TR. Figure 8 shows images acquired in a 42 year-old male referred for CMR to characterize a large inter-atrial mass seen by echocardiography. The phase-modulated 1-(90°)-1 WE-SSFP suppressed signal in the mass (Additional file 1), which had high signal in conventional cine-SSFP (Additional file 2), providing evidence supporting that the mass was a lipoma, precluding the need for further invasive diagnostic procedures.

## Discussion

We have shown that the simple combination of a phase-modulated 1-(90°)-1 water-excitation pulse together with cine-SSFP results in a fat suppressed steady-state with only minimal increase in TR and overall scan time. This technique utilizes the frequency offset between fat and water spins and a binomial pulse design to effectively suppress the normally bright fat signal in cine-SSFP. As shown by Thomasson *et al*. [21], the component pulse spacing in binomial water excitation need not be restricted to the time necessary to allow 180 degrees of phase evolution between fat and water. By appropriate RF phase modulation, component pulse spacing can be shortened while maintaining fat suppression. The resultant rapid water-excitation pulses incur only a minimal increase in TR, critical in cine-SSFP to avoid off-resonance banding and blood flow artifacts. Results in phantoms showed that the fat suppression achieved is similar to that predicted by Bloch equation simulations (Figures 2, 3 and 4), and *in vivo *results showed that this technique can significantly reduce bright fat signal while maintaining SSFP image quality (Figures 5, 6, 7, and 8). Furthermore, Figures 2d, h and 2l show a single-sided stop-band for the 1-(90°)-1 pulse at -220 Hz (i.e. the fat frequency) instead of the double-sided stop bands at ± 220 Hz (Figures 2b, f, j and 2c, g, k) demonstrated by the other WE pulses. The single-sided stop band may be an advantage as it is less likely to lead to suppression of water signal in case of field inhomogeneity. The frequency response profiles in Figure 3 demonstrated that the stopband frequency of the 1-(90°)-1 binomal pulse is independent on the choice of TR. Moreover, the 1-(90°)-1 pulse demonstrated consistent fat suppression at different TR's in water and mineral oil phantoms (Figure 4d–f). Phantom and *in vivo* signal measurements showed consistent fat signal attenuation was achieved without restriction of TR.

Existing fat suppression methods that have been described for SSFP applications [7-10,12,13] are generally of limited use in breath-hold SSFP cine imaging because they entail prolonged acquisition time, increased TR, or disruption of the steady-state. WE-SSFP has significant similarities with the fat suppressed alternating repetition time (FS-ATR) technique described by Leupold et al. [11]. The difference between the techniques is primarily conceptual, and both show that fat suppression can be achieved while maintaining the steady-state with only a minimal (~30%) increase in TR. Leupold describes frequency response and fat suppression in terms of a new steady-state defined by the alternation of TR between excitation pulses, and places certain restrictions on the relationship between the two TR's. Specifically, Leupold states that a TR = 4.3 ms is necessary for fat suppression at 1.5T. However, he goes on to show that fat suppression can still be achieved to some degree while allowing TR to vary. Our approach instead recognizes that the water-excitation pulse can be defined as a phase-modulated 1-(90°)-1 binomial pulse pair independent of other imaging sequence parameters. Based on this, we provide a simplified description of the method, and avoid unnecessary restrictions on the sequence design. The WE-SSFP technique described here imposes no specific restrictions on TR other than the usual SSFP requirement that TR<<T2, and no fixed relationship between TR and binomial pulse spacing. While TR must be increased to accommodate the binomial pulse length, the flexibility of choice in binomial WE pulse design and selection of imaging TR was demonstrated in the phantom and *in vivo* results. Three different configurations of WE pulses and TR values ranging from 4.0 ms to 8.9 ms were shown. The time between the component pulses of the binomial pulse series can be flexibly chosen based on slice profile and gradient constraints, with the understanding that lengthening the overall TR can have adverse effects on SSFP image quality. Any increase in TR in SSFP increases sensitivity to field inhomogeneity and flow.

One important limitation of this phase-modulated 1-(90°)-1 WE method is that field inhomogeneities can cause non-uniform fat suppression. However, this is true of any frequency-selective fat suppression scheme, and initial results in human subjects show sufficient homogeneity that these effects are not severe at 1.5T. The variability in fat suppression throughout the field-of-view observed in the *in vivo* images acquired with different pulses may be due to a variety of factors. As shown in Figure 2 and Figure 3, the frequency response pattern varies from pulse to pulse, and also with TR. Since these are breath-hold images of a beating heart, there can be variation in position causing variation in local homogeneity from one scan to the next. These factors may all contribute to the observed differences.

Another consideration is that phase-modulated 1-(90°)-1 WE increases specific absorption rate (SAR) at a given effective flip angle when compared to standard SSFP or binomial WE with 180° phase evolution. When 180° of phase evolution is allowed, each component pulse fully serves to further tip the water signal towards the transverse plane; the total resultant flip angle is divided evenly between the two α° pulses in a 1-(180°)-1 binomial pulse. However, in phase-modulated 1-(90°)-1 binomial WE, the tipping of water into the transverse plane is accomplished almost entirely by the first pulse, while the second pulse serves primarily to tip fat back up to the longitudinal axis. This is illustrated in Figure 9, which shows the resultant flip angle as a function of the individual component pulse flip angles for the 1-(90°)-1 pulse; the resultant flip angle is less than the sum of the flip angles of the component pulses. As a result, the SAR is increased relative to the conventional, single pulse selective excitation. The ratio of SAR for the 1-(90°)-1 WE pulse to the SAR for the conventional single pulse is also plotted in Figure 9. For example, the SAR is increased by about 20% (SAR ratio = 1.2) compared to the conventional pulse at an effective flip angle of 70°. This could be a significant limitation in the application of this technique at higher field strengths, although it could potentially be overcome by allowing longer phase evolution (greater delay time) between the component pulses. It would also be possible to lengthen the component RF pulses to reduce SAR without extending the total binomial pulse duration by using a bi-polar rather than mono-polar slice-selective gradient waveform. By alternating the polarity of slice selection gradient pulses from one component pulse to the next, the additional gradient lobes required for refocusing can be eliminated. However, the gradient first moment and therefore velocity sensitivity are greatly increased, and preliminary testing of this type of pulse design resulted in increased flow artifacts.

## Conclusion

In conclusion, our results show fat suppression is feasible by the combination of phase modulated binomial water excitation with SSFP cine CMR. It was found that a phase-modulated RF slice-selective pulse with phase evolution equal to 90° (1.1 ms interpulse delay) is sufficient to null fat signal while maintaining steady-state equilibrium for high SNR, insensitivity to off-resonance artifacts, and time-efficiency. Further testing is warranted to evaluate the effectiveness of this technique in clinical imaging.

## Competing interests

This research is partially supported by a grant from Siemens Healthcare, Inc.

## Authors' contributions

H–YL, Y–CC, and OPS all contributed to data collection, data analysis, and manuscript preparation. SVR contributed to data collection, data interpretation, and manuscript review. All authors approved the final version of the manuscript submitted.

## Supplementary Material

Additional file 1 Fat-suppressed WE-SSFP cine movie loop in patient with large intracardiac lipoma. Click here for file

Additional file 2 SSFP cine movie loop in patient with large intracardiac lipoma. Click here for file
