# Supplementary figures and images for: Rapid phase-modulated water excitation steady-state free precession for fat suppressed cine cardiovascular MR
Source: J Cardiovasc Magn Reson. 2008 May 13;10(1):22. doi: 10.1186/1532-429X-10-22 (PMC2429911; doi:10.1186/1532-429X-10-22)

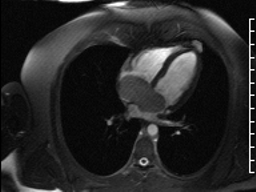

Supplement: Additional file 1 — Fat-suppressed WE-SSFP cine movie loop in patient with large intracardiac lipoma. [file 1532-429X-10-22-S1.gif]

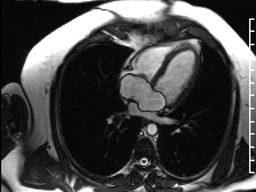

Supplement: Additional file 2 — SSFP cine movie loop in patient with large intracardiac lipoma. [file 1532-429X-10-22-S2.gif]
